# Somatic Evolution in Non-neoplastic IBD-Affected Colon

**DOI:** 10.1016/j.cell.2020.06.036

**Published:** 2020-08-06

**Authors:** Sigurgeir Olafsson, Rebecca E. McIntyre, Tim Coorens, Timothy Butler, Hyunchul Jung, Philip S. Robinson, Henry Lee-Six, Mathijs A. Sanders, Kenneth Arestang, Claire Dawson, Monika Tripathi, Konstantina Strongili, Yvette Hooks, Michael R. Stratton, Miles Parkes, Inigo Martincorena, Tim Raine, Peter J. Campbell, Carl A. Anderson

**Affiliations:** 1 Wellcome Sanger Institute, Hinxton, Cambridgeshire CB10 1SA, UK; 2 Department of Hematology, Erasmus University Medical Center, Postbus 2040, 3000 CA Rotterdam, the Netherlands; 3 Department of Gastroenterology, Addenbrooke’s Hospital, Cambridge University Hospitals NHS Foundation Trust, Hills Road, Cambridgeshire CB2 0QQ, UK; 4 Department of Histopathology, Cambridge University Hospitals NHS Foundation Trust, Hills Road, Cambridgeshire CB2 0QQ, UK; 5 University of Cambridge, Department of Paediatrics, Cambridge CB2 0QQ, UK

**Keywords:** inflammatory bowel disease, somatic mutations, mutation rate, Crohn's disease, ulcerative colitis, mutational signatures, intestinal epithelia, PIGR, IL17, ZC3H12A

## Abstract

Inflammatory bowel disease (IBD) is a chronic inflammatory disease associated with increased risk of gastrointestinal cancers. We whole-genome sequenced 446 colonic crypts from 46 IBD patients and compared these to 412 crypts from 41 non-IBD controls from our previous publication on the mutation landscape of the normal colon. The average mutation rate of affected colonic epithelial cells is 2.4-fold that of healthy colon, and this increase is mostly driven by acceleration of mutational processes ubiquitously observed in normal colon. In contrast to the normal colon, where clonal expansions outside the confines of the crypt are rare, we observed widespread millimeter-scale clonal expansions. We discovered non-synonymous mutations in *ARID1A*, *FBXW7*, *PIGR*, *ZC3H12A*, and genes in the interleukin 17 and Toll-like receptor pathways, under positive selection in IBD. These results suggest distinct selection mechanisms in the colitis-affected colon and that somatic mutations potentially play a causal role in IBD pathogenesis.

## Highlights

• IBD-affected colons accrue substitutions and indels 2.4 and 7 times faster than normal
• 17 signatures of mutational processes, including treatment
• Millimeter-scale clonal expansions late in molecular time
• Distinct mechanisms of positive selection of mutations in immune-related genes

## Graphical Abstract

**Figure:**
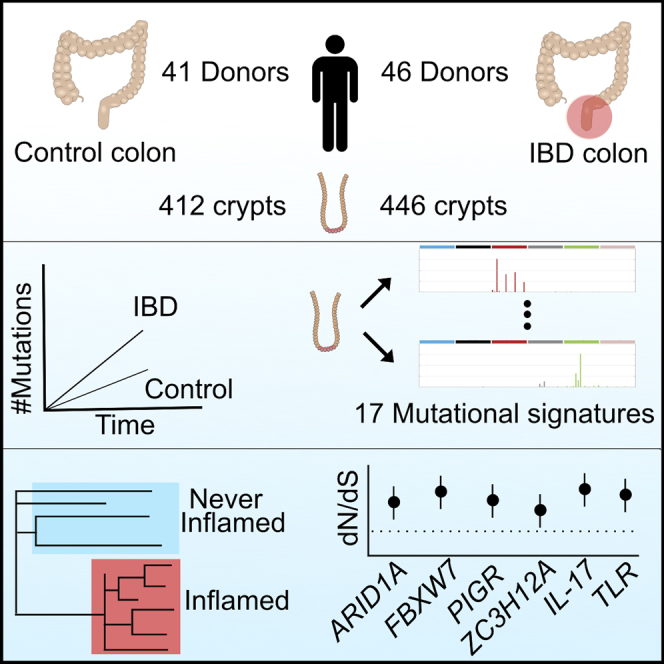

## Teaser

Whole-genome sequencing of inflammatory bowel disease patient samples allows insight into mutational burdens and processes associated with disease, including putative driver mutations positively selected in the diseased colon.

## Introduction

Inflammatory bowel disease (IBD) is a debilitating disease characterized by repeated flares of intestinal inflammation. The two major subtypes of IBD, Crohn’s disease (CD) and ulcerative colitis (UC), are distinguished by the location, continuity, and nature of the inflammatory lesions. UC affects only the large intestine, spreading continuously from the distal to proximal colon, whereas CD most commonly affects the small and large intestine and is characterized by discontinuous patches of inflammation. In addition to the significant morbidity associated with the disease, IBD patients have a 1.7-fold increased risk of developing gastrointestinal cancers compared to the general population. Cancer risk is associated with the duration, extent, and severity of disease, and cancers tend to occur earlier in life in IBD patients (Lutgens et al., 2013; Beaugerie and Itzkowitz, 2015; Adami et al., 2016). As a result, patients require regular endoscopic screening and may undergo prophylactic colectomy to mitigate this risk (Beaugerie and Itzkowitz, 2015; Adami et al., 2016).

That somatic mutations contribute to the development of cancer is well established, but their patterns, burden, and functional consequences in diseases other than cancer have not been extensively studied. Methodological developments have now enabled the analysis of polyclonal somatic tissues, allowing characterization of somatic mutations in normal tissues such as skin (Martincorena et al., 2015), esophagus (Martincorena et al., 2018; Yokoyama et al., 2019), endometrium (Moore et al., 2020; Suda et al., 2018), lung (Yoshida et al., 2020), and colon (Blokzijl et al., 2016; Lee-Six et al., 2019). In the setting of non-neoplastic diseases, chronic liver disease has had the most attention, with studies showing that compared to healthy liver, hepatic cirrhosis is associated with acquisition of new mutational processes, increased mutation burden and larger clonal expansions (Brunner et al., 2019; Kim et al., 2019; Zhu et al., 2019).

Colonic epithelium is well suited to the study of somatic mutations because of its clonal structure. It is organized into millions of colonic crypts, finger-like invaginations composed of ∼2,000 cells (Potten et al., 1992) each extending into the lamina propria below. At the base of each crypt reside a small number of stem cells undergoing continuous self-renewal through stochastic cell divisions (Lopez-Garcia et al., 2010; Snippert et al., 2010). As a result, the progeny of a single stem cell iteratively sweeps through the entire niche, and the epithelial cells that line the crypt are the progeny of this single clone. Active IBD disrupts these normal stem cell dynamics—the epithelial lining is damaged, the organized crypt structure is ablated, and the barrier between lumen and mucosa is disrupted.

We hypothesized that the recurrent cycles of inflammation, ulceration, and regeneration seen in IBD could impact the mutational and clonal structure of intestinal epithelial cells. To test these hypotheses, we isolated and whole-genome sequenced 446 colonic crypts from 46 IBD patients with varying degrees of colonic inflammation, both active and previous, and compared the mutation burden, clonal structure, mutagen exposure, and driver mutation landscape to colonic crypts from healthy donors (Lee-Six et al., 2019).

## Results

### IBD More Than Doubles the Mutation Rate of Normal Colonic Epithelium

**Figure S1:**
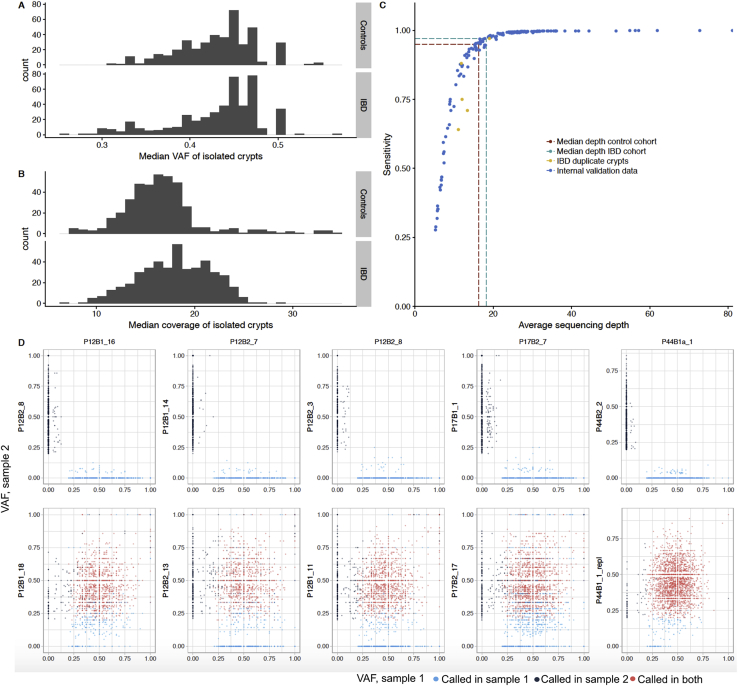
Clonality, Coverage, and Sensitivity of Crypts and Mutations Calls, Related to Figure 1 (A) The median variant allele fraction (VAF) of mutations called in each crypt. (B) The median coverage of sequenced crypts. (C) Internal analysis of CaveMan sensitivity. The dashed lines show interpolation of the sensitivity given the median coverage of cases (18.2X - 97% sensitivity) and controls (16.3X - 95% sensitivity). The yellow dots represent biological duplicates where sensitivity was estimated by dissecting and sequencing the same crypts twice (STAR Methods). (D) VAFs of variants called in crypts that were sequenced twice (referred to as sample 1). Each dot represents a variant. The VAFs are compared against variants called in unrelated crypts (top) and in biological duplicates (bottom). The high concordance between biological duplicates but not between unrelated samples suggests high specificity.

We used laser capture microdissection (LCM) to isolate 446 colonic crypts from endoscopic biopsies taken from 28 UC patients and 18 CD patients (Table S1). Biopsies were annotated as never, previously, or actively inflamed at the time of sampling (STAR Methods). The dissected crypts were whole-genome sequenced to a median depth of 18.2X, allowing us to call somatic substitutions and small insertions and deletions (indels) with high specificity and sensitivity (Figure S1; STAR Methods). We also called larger copy number changes, somatic retrotranspositions, and aneuploidies affecting whole chromosomes or chromosome arms (Tables S2 and S3; STAR Methods).

**Figure 1:**
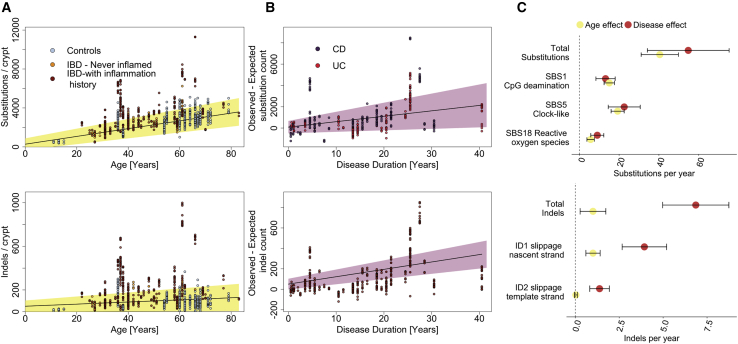
Mutation Burden in the IBD Colon (A) Substitution (top) and indel (bottom) burden as a function of age. Each point represents a colonic crypt and is colored by disease status. The line shows the effect of age on mutation burden as estimated by fitting a linear mixed effects model, correcting for sampling location, sequencing coverage, and the within-biopsy and within-patient correlation structure, considering both IBD cases and controls. The shaded area represents the 95% confidence interval of the age effect estimate. (B) Estimated excess of substitutions (top) and indels (bottom) in crypts from IBD patients as function of disease duration. Shaded area represents the 95% confidence interval. (C) A comparison of the effects of age and disease duration on the total mutation burden and on the burden of mutational signatures that associate with IBD duration. Error bars represent the 95% confidence intervals of the estimates. IBD, inflammatory bowel disease; CD, Crohn’s disease; UC, ulcerative colitis; SBS, single base substitution signature; ID, indel signature. See also Tables S1 and S2.

To assess if IBD is associated with a difference in the mutation burden of the colonic epithelium, we combined our data with data from 412 crypts sequenced as part of our recent study of somatic mutations in normal colon (Lee-Six et al., 2019) (hereafter referred to as the control data). We fitted linear mixed-effects models (LMMs) to estimate the independent effects of age, disease duration, and biopsy location on mutation burden, while controlling for the within-patient and within-biopsy correlations inherent in our sampling strategy (STAR Methods). Disease duration was included in the model as a proxy for inflammation exposure. We estimated the effect of IBD to be 55 substitutions per crypt per year of disease duration (35–75 95% confidence interval [CI], p = 3.1 × 10^−7^, LMMs and likelihood ratio test; Figure 1). These mutations are in addition to the 40 (31–50, 95% CI) substitutions we estimated are accumulated on average per year of life under normal conditions, suggesting that mutation rates are increased ∼2.4-fold in regions of the IBD-affected colon on average. Compared to controls, patients with IBD had greater between-patient variance in mutation burden (SD = 776 versus 383 substitutions and SD = 80 versus 34 indels for cases and controls, p = 4.2 × 10^−8^ and p = 1.1 × 10^−16^, respectively; LMMs and likelihood ratio test) and greater within-patient variance (SD = 955 versus 407 substitutions and SD = 81 and 18 indels for cases and controls, p = 0.032 and p = 0.0011, respectively). The increased between-patient variance likely reflects differences in inflammation exposure not captured by disease duration, because it does not account for variable disease severity, response to treatment, etc. among patients. The increased within-patient variance probably reflects region-to-region differences in disease severity along the colon. We similarly estimated an increase in the indel burden in IBD, with an excess of 6.8 indels per crypt per year of IBD (5.0—8.7 95% CI, p = 5.7 × 10^−11^; Figure 1) in addition to the estimated 1.0 (0.3–1.7 95% CI) indel that is accumulated per crypt per year of life. As shown in Figure 1B, a handful of clones and patients had a much higher mutation burden than expected given their age. This is partially driven by the effect of smoking and cancer driver status, as discussed below. The effect of IBD on the mutation burden remains significant if crypts carrying driver mutations, or the five IBD patients with the highest mutation burdens, are excluded (p = 0.0014 and 0.0099 for substitutions and p = 6.8 × 10^−6^ and 1.1 × 10^−5^ for indels, respectively). We found no significant difference in the mutation burden between UC and CD patients.

Smoking status was available for a subset of the IBD cohort (35 patients who contributed 362 crypts). In this restricted dataset, we found an effect of smoking duration of 49 (18–81 95% CI, p = 0.0024) substitutions and 5.3 (2.3–8.2 95% CI, p = 6.5 × 10^−4^) indels per crypt per year of smoking. The effect of disease duration was unchanged, suggesting this effect is not driven by differences in smoking habits between cases and controls. Smoking has been reported to increase the risk of CD and be protective for UC (Mahid et al., 2006), but we found no interaction effect between smoking and disease type (p = 0.68). Smoking status was not available for the control cohort. The full details of all LMMs are available in a GitHub directory accompanying this submission (https://github.com/Solafsson/somaticIBD/).

### IBD Accelerates Age-Related Mutational Processes

**Figure S2:**
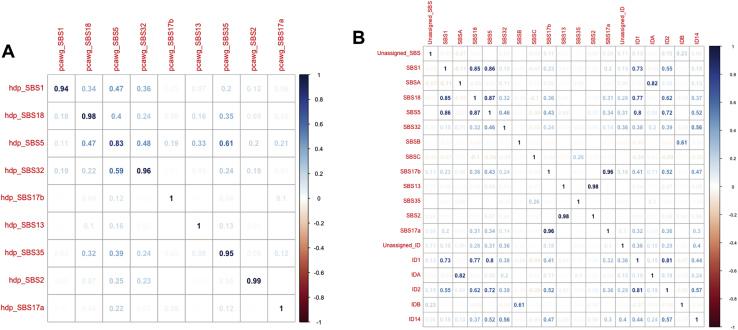
Features of Mutational Signatures Extracted, Related to Figure 2 (A) Cosine-similarities between mutational signatures extracted by hdp compared with published PCAWG signatures. (B) Correlations between identified signatures.

The somatic mutations found in the cells of a colonic crypt reflect the mutational processes that have acted on the stem cells and their progenitors since conception. Distinct mutational processes each leave a characteristic pattern, a mutational signature, within the genome, distinguished by the specific base changes and their local sequence context (Alexandrov et al., 2013, 2020). We extracted mutational signatures jointly for IBD and control crypts and discovered 12 substitution signatures (SBS) and five indel signatures (ID), all of which have been previously observed in tissues from individuals without IBD (Figure S2; Table S2; STAR Methods). Comparing our IBD cases and controls, we found that ∼80% of the increase in mutation burden in cases is explained by signatures that are also found ubiquitously in normal colon (Blokzijl et al., 2016; Lee-Six et al., 2019). These are substitution signatures 1, 5, and 18 and indel signatures 1 and 2, as defined by Alexandrov et al. (2020) (Figure 1), which cause an increase of 13 (8–18 95% CI), 23 (15–30 95% CI), and 9 (6–12 95% CI) substitutions per crypt per year of disease, respectively (p = 2.4 × 10^−7^, 1.0 × 10^−7^, and 3.2 × 10^−7^), and 4.3 (3.3–5.4 95% CI) and 1.7 (1.1–2.3, 95% CI) indels per crypt per year, respectively (p = 4.0 × 10^−12^ and p = 9.5 × 10^−8^, LMMs and likelihood ratio tests, see https://github.com/Solafsson/somaticIBD/ for full details of the models). Substitution signatures 1 and 5 are clock-like and thought to be associated with cell proliferation, whereas signature 18 has been linked with reactive oxygen species (Alexandrov et al., 2020). The indel signatures ID1 and ID2 are both thought to be the result of polymerase slippage during DNA replication (Alexandrov et al., 2020).

**Figure 2:**
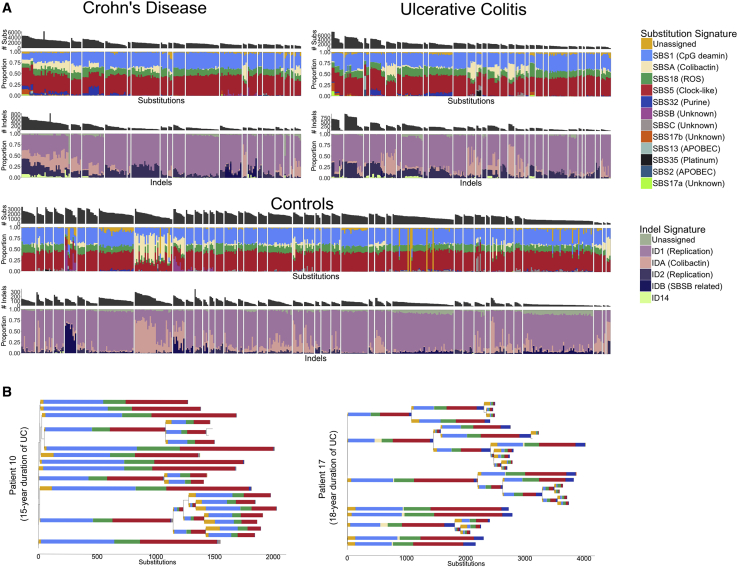
Mutational Signatures in Colonic Crypts (A) A stacked barplot showing the proportional contribution of single-base-substitution (SBS) signatures (top) and indel (ID) signatures (bottom) to the mutation burden of each crypt. Crypts are grouped by patient and crypts from CD, UC, and controls are shown separately. Signature nomenclature is the same as in Alexandrov et al. (2020). The “Unassigned” component represents uncertainty of the signature extraction. (B) Phylogenetic trees of two patients with widespread ulcerative colitis. The colors of the branches reflect the relative contribution of each mutational signature extracted for those branches as in (A). The patient on the left has received azathioprine treatment for 10 years but shows no SBS32 burden (dark blue). In contrast, the patient on the right received azathioprine for 2 weeks and mercaptopurine for 2 weeks and had significant adverse reactions to both drugs. SBS32 is found in most crypts from this patient. All crypts are from inflamed biopsies. See also Figure S2 and Table S2.

The remaining 20% of the increase in substitution burden is a consequence of rarer mutational processes and treatment. For example, 96 crypts had over 150 mutations attributed to purine treatment in a subset of seven IBD patients, five of whom have a documented history of such treatment. However, the number of mutations attributed to purine was not associated with purine therapy duration, and some patients showed large mutation burdens despite brief, or indeed no, documented exposure. For example, one patient received azathioprine for 2 weeks and mercaptopurine for 2 weeks and had significant adverse reactions to both drugs. This brief treatment resulted in a median of 204 mutations (range: 120–374) attributed to purine treatment in the crypts from this individual. Other patients had long-term exposure to azathioprine without accruing any purine-related mutations (Figure 2B). There was also great within-patient variation in the burden of the purine signature. The largest range was observed for patient 40, which has a 7 year history of purine treatment. The estimated burden of the purine signature in crypts from this patient ranged from 69–1,005. The reason for this large variation in purine-related mutation burden remains unknown, although it is associated with the number of putative cancer drivers (see below). Thiopurine use has been associated with higher overall cancer risk in epidemiological studies, but this is mostly driven by an effect on lymphoid cancers and possibly on urinary tract cancers, but not colorectal cancers (Pasternak et al., 2013; Adami et al., 2016). The relationship between purines and colon cancer is complicated and requires further study. On one hand, our results show purine-related mutations accumulating in the crypts of a subset of patients, but on the other hand, effective purine treatment may prevent disease-related mutagenesis.

**Figure S3:**
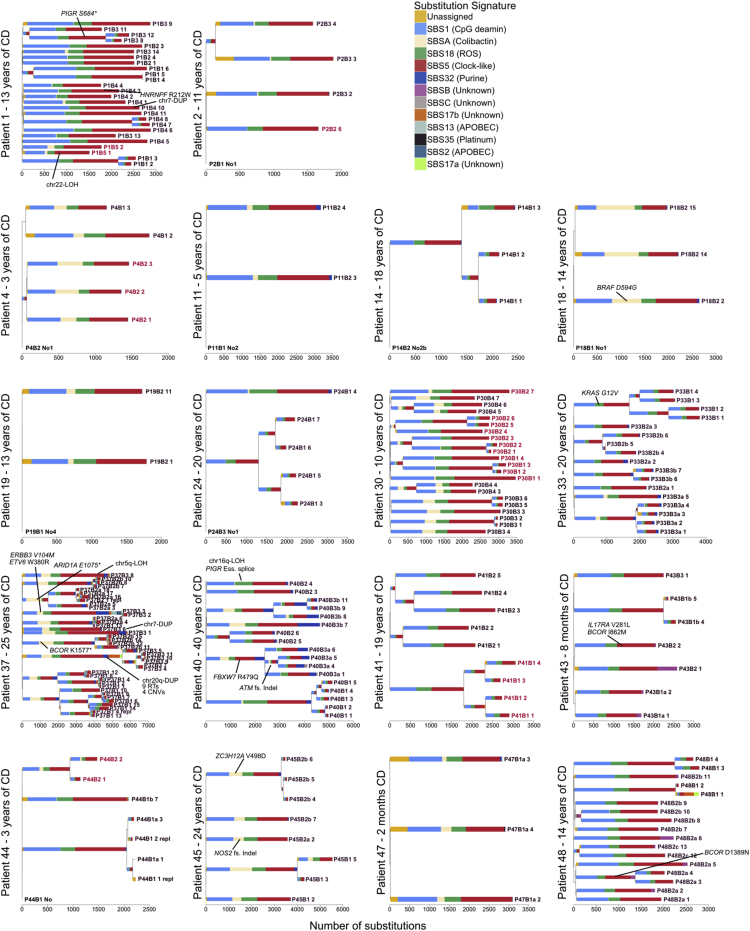
Phylogenetic Trees for All Crohn’s Disease Patients, Related to Figures 2, 4, and 6 Mutational signatures are overlaid on the trees and putative driver mutations are mapped to the branch in which they occur. Crypts are labeled on the form PXBY_Z where PX is the patient number, BY the biopsy number (with a,b and c denoting biopsies taken a few millimeters apart from the same site) and Z is the crypt number. The crypts labeled in red are from never-inflamed regions of the colon.

**Figure S4:**
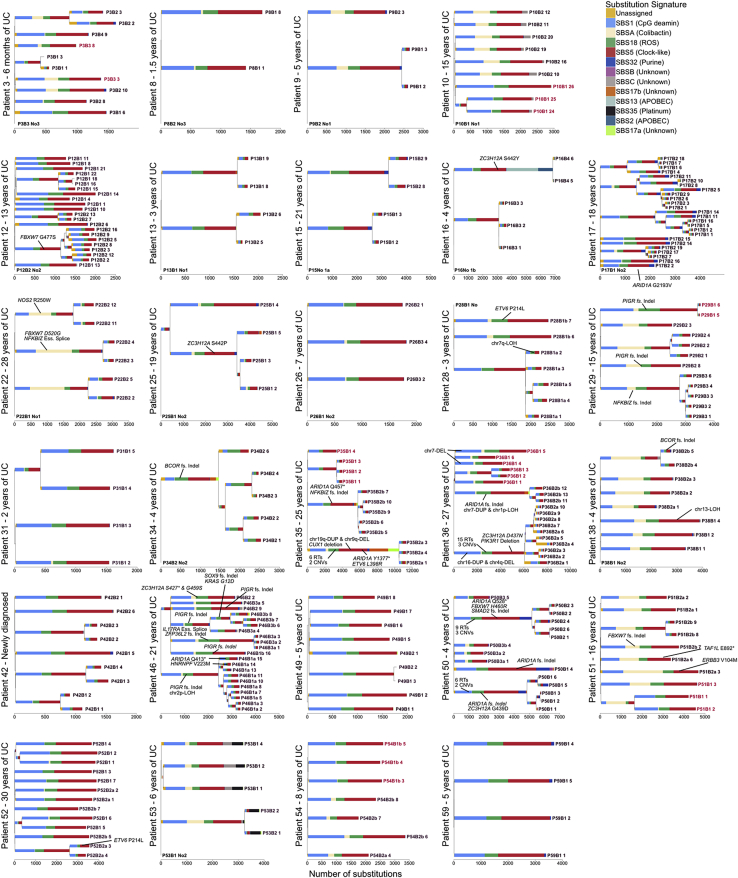
Phylogenetic Trees for All Ulcerative Colitis Patients, Related to Figures 2, 4, and 6 Mutational signatures are overlaid on the trees and putative driver mutations are mapped to the branch in which they occur. Crypts are labeled on the form PXBY_Z where PX is the patient number, BY the biopsy number (with a,b and c denoting biopsies taken a few millimeters apart from the same site) and Z is the crypt number. The crypts labeled in red are from never-inflamed regions of the colon.

Five signatures previously discovered in the normal colon (Lee-Six et al., 2019), SBSA, SBSB, SBSC, IDA, and IDB were also present in the context of IBD. SBSA and IDA and SBSB and IDB are highly correlated (Figure S2B) and likely represent the same underlying mutational processes. SBSA and IDA are of particular interest because they have recently been shown to be caused by the genotoxin colibactin, which is produced by bacteria harboring a polyketide synthases (pks) pathogenicity island (Pleguezuelos-Manzano et al., 2020). *pks*^+^ *E. coli* have been reported at increased frequency in IBD (Arthur et al., 2012), but we found no relationship between SBSA or IDA burden and disease status or disease duration after correcting for higher burden of both in the left-side of the colon (the site primarily affected in UC). As in normal colon, SBSA and SBSB were primarily found in early branches of the phylogenetic trees (Figures S3 and S4). Signatures SBSB, SBSC, and SBS32 have not been reported in studies of sporadic colorectal cancers (Alexandrov et al., 2020), perhaps due to the comparative complexity and diversity of cancer mutation profiles. SBS32, however, would only be expected in patients receiving purine therapy and so would not be present in sporadic colorectal cancers. These signatures have also not been reported in studies of colitis-associated colorectal cancers but this is likely due to a relative lack of power due to the small number of sequenced exomes (Robles et al., 2016; Baker et al., 2019; Din et al., 2018).

**Figure S5:**
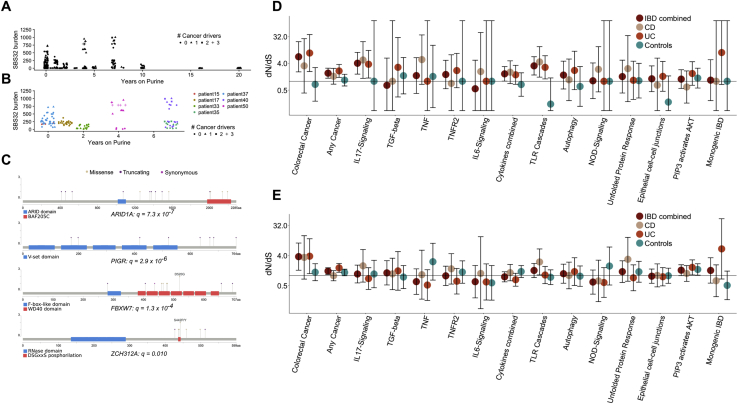
Driver Mutations and Positive Selection, Related to Figure 6 (A) Burden of the purine-signature, SBS32, as a function of the duration of purine treatment. (B) Burden of the purine-signature in patients where at least one crypt has over 150 mutations attributed to purine treatment. Large within-patient variation is apparent. (C) A lollipop plot showing the location of mutations found in genes that are enriched for non-synonymous coding mutations in the IBD dataset. (D) Pathway-level dN/dS ratios for mutations in known cancer genes and cellular pathways important in IBD pathogenesis. The plot shows dN/dS for truncating mutations. Same as Figure 6C but also showing the ratios for controls and ratios obtained when analysis is restricted to Crohn’s disease or ulcerative colitis crypts. Error bars represent 95% confidence intervals. (E) same as (D) but showing dN/dS ratios for missense mutations.

Signatures 2 and 13, which are associated with APOBEC activity, and signatures 17a and 17b, which are of unknown etiology, were active in a small number of crypts with high mutation burdens. SBSB, SBSC, SBS17a/b, and SBS2/SBS13 are too rare for us to be powered to detect any difference between IBD and controls or to associate these with any clinical feature documented in our metadata. Finally, we found signature 35, associated with platinum compound therapy, in one patient with a history of platinum treatment for squamous cell carcinoma of the tongue. The patient received 40 mg/m^2^ of cisplatin therapy on a weekly basis. He completed three of six planned treatment cycles with therapy termination due to toxicity. This relatively brief treatment resulted in a medium of 430 mutations (range: 350–461) per crypt that were attributed to signature 35, equivalent to ∼10 years of normal mutagenesis. In crypts from this individual, we also observed a small number of double base substitutions of CT > AA and CT > AC classes (median 6, range: 5–13), probably indicative of DBS5, which has also been linked with platinum treatment.

### IBD Associates with the Burden of Structural Variants

**Figure 3:**
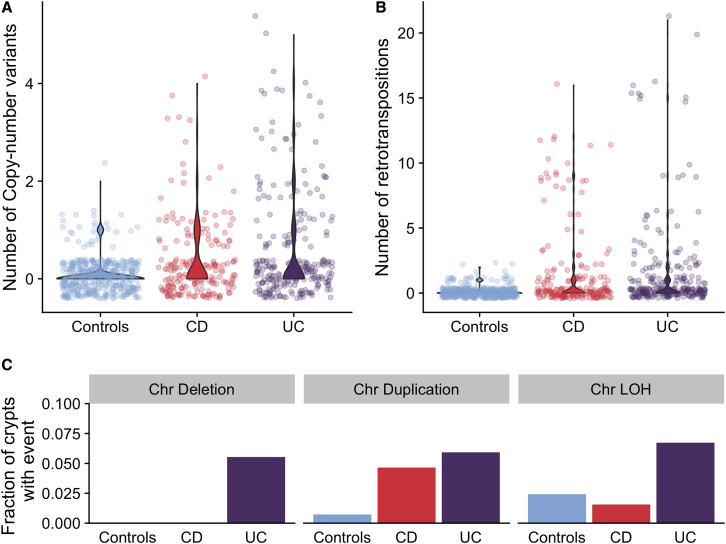
Burden of Structural Variants in Inflammatory Bowel Disease-Affected Colon Compared with IBD-Unaffected Colon (A) Number of copy number variants in IBD sub-types compared with controls. (B) Number of somatic retrotranspositions in IBD subtypes compared with controls. (C) Fraction of crypts with inflammation history that carry chromosomal aneuploidies. See also Table S3.

We called copy number variants (CNVs), somatic retrotranspositions, and loss-of-heterozygosity events affecting whole chromosomes or chromosome arms (referred to here as aneuploidies for simplicity) in both our IBD cases and non-IBD controls. The burden of structural variants is modest in both datasets (Figure 3), but for IBD, we identified the occasional clone that carried a large number of CNVs and retrotranspositions (Figures 3A and 3B). The numbers of CNVs and retrotranspositions are associated with IBD duration. We estimated the CNV mutation rate to be 0.067 CNVs per crypt per year of disease (0.027–0.11 95% CI, p = 1.1 × 10^−3^, likelihood ratio test of mixed-effects Poisson regressions) and the retrotransposition mutation rate to be 0.065 (0.018–0.11, 95% CI, p = 6.9 × 10^−3^). This corresponds to one CNV per crypt every 14.9 years of disease duration and one retrotransposition event every 15.4 years of disease duration on average. However, a handful of clones accumulated many structural variants (SVs), whereas the majority had none, suggesting that the processes driving their acquisition may be episodic rather than continuous. This would be in line with findings from other reports linking rapid accrual of SVs with the transition from normal to dysplastic mucosa (Baker et al., 2019) and cancers accruing copy number gains in a punctuated manner (Gerstung et al., 2020).

We found a higher fraction of IBD crypts carrying aneuploidies than in controls (43/419 compared with 13/412, Figure 2C). However, this was driven by large clones carrying aneuploidies, and the number of events was not significantly associated with disease duration (p = 0.42). No type of chromosomal abnormality differed significantly between UC and CD (p = 0.65, 0.83, and 0.67 for deletions, duplications, and loss-of-heterozygosity, respectively; binomial mixed effects model). The numbers of CNVs, retrotranspositions, and aneuploidies are associated with higher substitution burden (112 [49–175 95% CI, p = 6.4 × 10^−4^], 59 [38–81 95% CI, p = 1.5 × 10^−7^], and 199 [65–331 95% CI, p = 3.7 × 10^−3^], respectively), and retrotranspositions and CNVs are associated with higher indel burden (11 [8–14 95% CI, p = 2.6 × 10^−12^] and 17 [10–24 95% CI, p = 6.7 × 10^−6^], respectively).

### IBD Creates a Patchwork of Millimeter-Scale Clones

**Figure 4:**
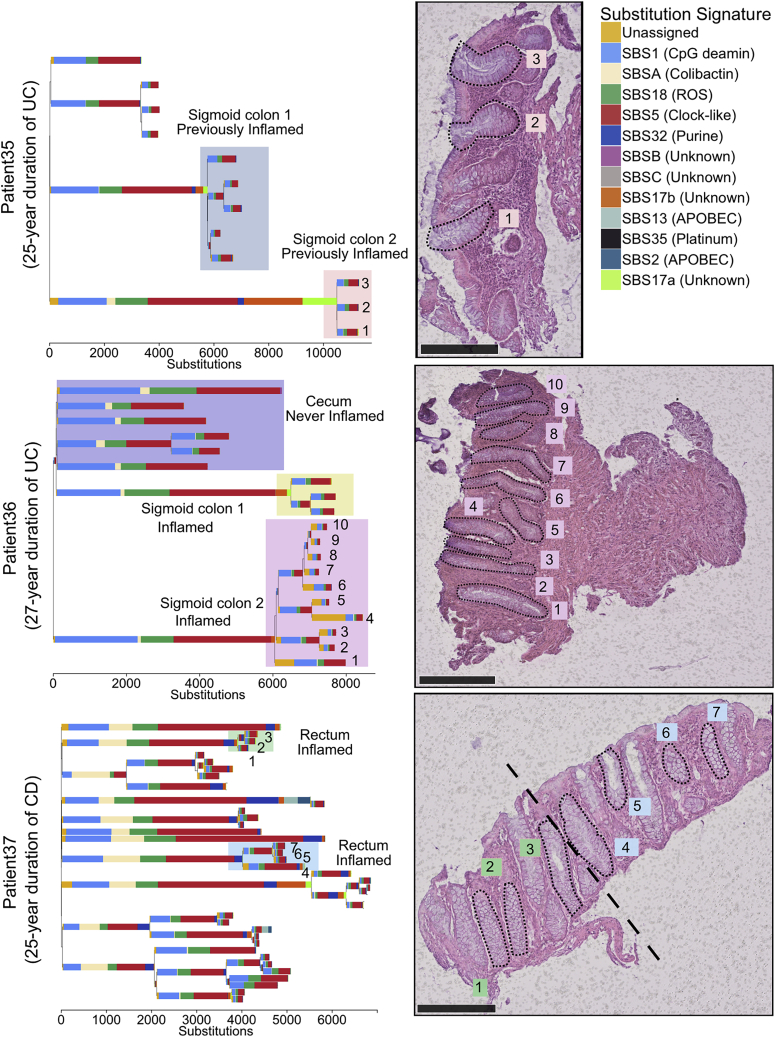
Examples of Clonal Expansions in Three IBD Patients Top: a phylogenetic tree of crypts sampled from a 66-year-old patient with a 25-year history of ulcerative colitis. The accompanying biopsy image shows the crypts from the orange shaded area. The clones highlighted in blue and orange come from the same previously inflamed site and were millimeters apart. A large difference in the mutation burden of these clones is driven by a local activation of signatures 17a and 17b in the orange shaded clone. Middle: a phylogenetic tree of crypts sampled from a 61-year-old patient with a 27-year history of ulcerative colitis. The clones highlighted in purple and yellow come from biopsies taken millimeters apart. The accompanying biopsy image shows the crypts from the purple clone. Bottom: a phylogenetic tree of crypts sampled from a 37-year-old patient with a 25-year history of Crohn’s disease affecting the colon. A biopsy overlaps two clones (in blue and green). Scale bars, 250 μm. See also Figures S3 and S4.

**Figure 5:**
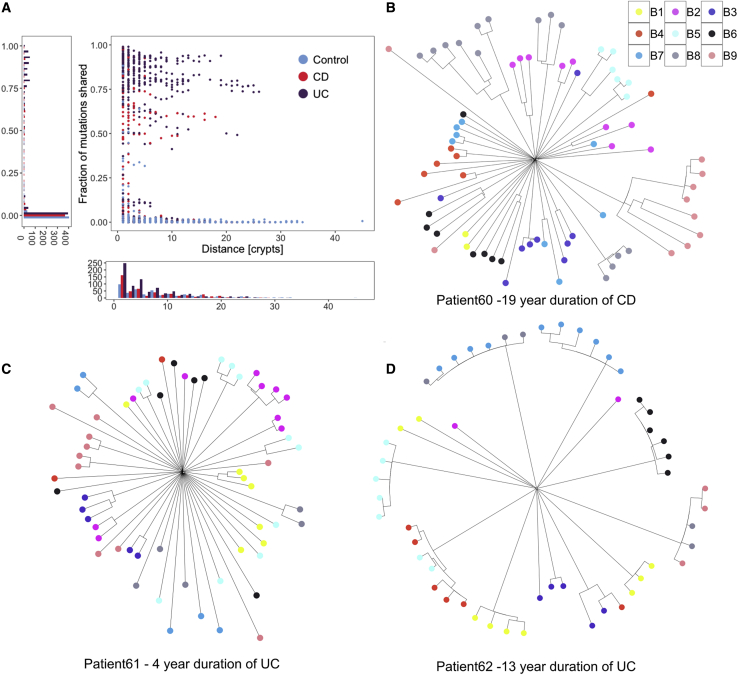
Clonal Structure of the IBD Colon (A) For pairs of crypts from the same biopsy, the figure shows the number of mutations that are shared between a pair as a fraction of the average mutation burden of the two crypts, and this is plotted as a function of the distance between the pair. (B) A phylogenetic tree showing crypts sampled from 9 biopsies from the sigmoid colon of a 36-year-old male diagnosed with Crohn’s disease 19 years prior to sampling. Biopsies were taken 1 cm apart in a three by three grid for all the patients in (B)-(D). (C) A phylogenetic tree showing crypts sampled from 9 biopsies from the rectum of a 71-year-old male diagnosed with ulcerative colitis 4 years prior to sampling. (D) A phylogenetic tree showing crypts sampled from 9 biopsies from the rectum of a 42-year-old female diagnosed with ulcerative colitis 13 years prior to sampling. See also Figures S3 and S4.

Colonic crypts divide by a process called crypt fission, whereby a crypt bifurcates at the base, and branching elongates in a zip-like manner toward the lumen. This process is relatively rare in the normal colon, wherein each crypt fissions on average only once every 27 years (Nicholson et al., 2018; Lee-Six et al., 2019). Compared to normal colon, we found much larger clonal expansions in IBD patients, evident of numerous crypt fission events occurring late in molecular time. We observed several examples of individual clones spanning entire 2–3 mm endoscopic biopsies (Figures 4, 5A, S3, and S4). Our ability to estimate clone sizes is restricted by the small size of the biopsies. However, when we biopsied the same inflamed or previously inflamed region more than once, on only one occasion out of 19 biopsy pairs did we observe a clone stretching between biopsies that were taken a few millimeters apart (Figures S3 and S4), whereas most biopsies contained more than one clone. To improve our ability to detect larger clones, we sampled three patients more broadly. Nine biopsies, forming a 3 × 3 grid with 1 cm separating biopsies, were obtained from each patient. We dissected 187 crypts from the biopsies and performed whole exome sequencing on individual crypts. Phylogenetic trees were reconstructed based on somatic mutations identified (Figure 5B–5D). Although clonal expansions within biopsies are common, we found clones extending between neighboring biopsies in only one of these patients. A substantial body of evidence exists documenting widespread clonal expansions giving rise to dysplasia and ultimately to colorectal cancer in IBD (reviewed in Choi et al., 2017). Colitis-associated colorectal cancers, which are enriched with synchronous lesions (Lam et al., 2014; Choi et al., 2015), commonly grow from a background of a pre-cancerous field that has expanded many centimeters or even the whole length of the colon (Leedham et al., 2009; Galandiuk et al., 2012). Mutations in *TP53* are thought to be especially prominent in the growth of these clones, but aneuploidies and *KRAS* mutations are also commonly observed (Holzmann et al., 1998; Leedham et al., 2009; Galandiuk et al., 2012). In our material of non-dysplastic tissue from individuals without colorectal neoplasia, we find smaller clones and mutations in *TP53*, *KRAS*, or *APC* are rare. In summary, IBD-affected regions are generally not dominated by a single major clone, but are more accurately viewed as an oligoclonal patchwork of clones that often grow considerably larger than in healthy colon.

### Distinct Patterns of Selection in IBD Compared with Normal Epithelium

The recurrent cycles of inflammation and remission that characterize IBD could create an environment in which clones containing advantageous mutations may selectively spread in the mucosa. This advantage may manifest either through faster cell division and elevated crypt fission rate or through increased resistance to the cytotoxic effects of inflammation. To identify mutations that likely confer selective advantage on the cell, we searched for mutations occurring in canonical mutation hotspots from the Cancer Genome Atlas (Table S4A). This revealed a total of 10 missense mutations in *KRAS*, *BRAF*, *TP53*, *ERBB2*, *ERBB3*, and *FBXW7* occurring at canonical hotspots (Table S4B). Additionally, we found a heterozygous nonsense mutation in *APC* and frameshift indels in known colorectal tumor suppressors; *ATM*, *SOX9*, *RNF43*, and *ZFP36L2*, of likely driver status (Figure 4A; Table S4B). Furthermore, two large-scale deletions in our dataset overlap known tumor suppressors, *PIK3R1* and *CUX1*, and are likely drivers. The number of putative cancer drivers found in a crypt is associated with increased burden of both substitutions (269 substitutions per driver, 90–447 95% CI, p = 5.6 × 10^−3^) and indels (40 indels per driver, 20–60 95% CI, p = 1.7 × 10^−4^), as well as with each of the replication-related signatures (SBS1, SBS5, SBS18, ID1, and ID2; Table S4C). There was also a significant association with the purine signature (SBS32). We estimated the burden of purine signature to be increased by 30 (14–47, 95% CI, p = 3.7 × 10^−4^; Figures S5A and S5B) substitution per driver, suggesting that rapidly dividing cells may be particularly susceptible to the mutagenic effect of purine treatment.

**Figure 6:**
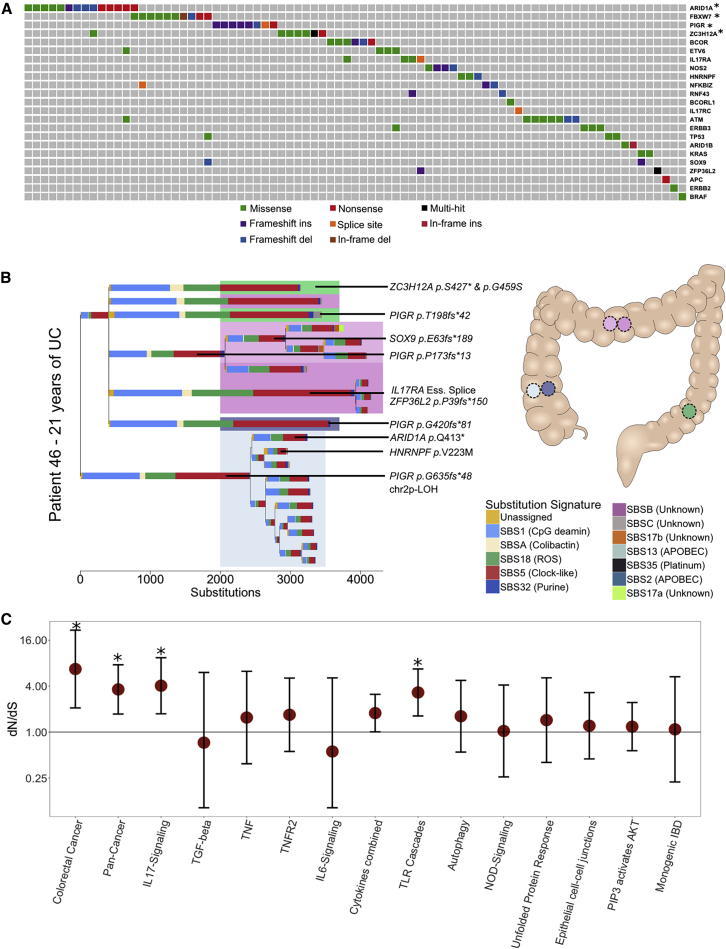
Driver Mutations and Positive Selection in IBD (A) An oncoplot showing the distribution of potential driver mutations mapped to branches of phylogenetic trees. Each column represents a branch of a phylogenetic tree and a mutation may be found in multiple crypts if the branch precedes a clonal expansion. Branches without potential drivers are not shown for simplicity. ^∗^Genes showing significant enrichment of non-synonymous mutations after Benjamini-Hochberg correction for multiple testing (q < 0.05). (B) A phylogenetic tree of the crypts dissected from a 38-year-old male suffering from ulcerative colitis for 21 years. Crypts are dissected from five biopsies from three previously inflamed sites of the colon. Crypts carrying distinct *PIGR* truncating mutations are found in four of the biopsies and in all three colonic sites. (C) Pathway-level dN/dS ratios for truncating mutations in known cancer genes and cellular pathways important in IBD pathogenesis. Error bars represent 95% confidence intervals. *q < 0.05 after Benjamini-Hochberg correction for multiple testing. See also Figure S5 and Tables S4, S5, and S6.

To search for genes under positive selection, we assessed the ratio of non-synonymous to synonymous mutations (dN/dS) across all IBD crypts, while correcting for regional and context-dependent variation in mutation rates (Martincorena et al., 2017). Genes with dN/dS ratios significantly different from 1 are considered to be under selective pressure. This analysis revealed four genes, *ARID1A*, *FBXW7*, *PIGR*, and *ZC3H12A*, to be under positive selection in the IBD colon (Figures 6A and S5C; Table S4B). *ARID1A* and *FBXW7* are well-established tumor suppressors and are found mutated at similar frequencies in sporadic- and colitis-associated colorectal cancers (Martincorena et al., 2017; Baker et al., 2019). In several instances, distinct heterozygous mutations in the same gene were found in different crypts from the same patient (Figures S3 and S4). For example, in one patient suffering from pan-colitis, we found four distinct *PIGR* mutations in four biopsies from the right, transverse, and left side of the colon (Figure 6B). We did not detect a significant signal of selection of mutation in the two genes, *AXIN2* or *STAG2*, which we previously found to be under positive selection in the normal colon (Lee-Six et al., 2019) (p = 0.98 and 0.74, respectively) nor was there any evidence of selection of *PIGR* or *ZC3H12A* mutants in the normal colon (Table S5B). We did not find a significant difference in the mutation burden of any of these genes between UC and CD, suggesting that similar selection pressures are operative in mucosal tissue in both diseases.

Recurrent mutations in *PIGR* and *ZC3H12A* are of particular interest because these have not been described in cancer but have roles in immunoregulation and reflect distinct mechanisms of positive selection in the IBD colon. *PIGR* encodes the poly-immunoglobulin (Ig) receptor, which transfers polymeric Igs produced by plasma cells in the mucosal wall across the epithelium to be secreted into the intestinal lumen (Johansen and Kaetzel, 2011). *Pigr* knockout mice exhibit decreased epithelial barrier integrity and increased susceptibility to mucosal infections and penetration of commensal bacteria into tissues (Johansen et al., 1999). *ZC3H12A* encodes an RNase, Regnase-1 (also known as *MCPIP1*). It is activated in response to TLR stimulation and degrades mRNA of many downstream immune signaling genes (Matsushita et al., 2009), including *PIGR* (Nakatsuka et al., 2018), *NFKBIZ* (Mino et al., 2015), and members of the interleukin-17 (IL-17) pathway (Garg et al., 2015). Four of the mutations in *ZC3H12A* occur in a DSGxxS motif that, when phosphorylated, marks the protein for ubiquitin-mediated degradation. Mutations of the corresponding residues in mice attenuate the phosphorylation (Iwasaki et al., 2011) and stabilize the protein so these are likely gain-of-function.

We next carried out a pathway-level dN/dS analysis, searching for enrichment of missense and truncating variants across 15 gene sets that were defined *a priori* because of their relevance in either colorectal carcinogenesis or IBD pathology (Figure 6C; Table S5; STAR Methods). We observed a 6.5-fold (1.8–23.6, 95% CI) enrichment of truncating mutations in genes associated with colorectal cancer (q = 0.011) as well as a 1.9-fold (1.3–2.8, 95% CI) enrichment in genes significant in a pan-cancer analysis of selection (Priestley et al., 2019) (q = 0.011). Interestingly, the pathway-level dNdS also revealed a 4.0-fold (1.7–9.4, 95% CI) enrichment of truncating mutations in the IL-17 signaling pathway (q = 0.011) and a 3.3-fold (1.6–6.7, 95% CI) enrichment in Toll-like receptor (TLR) cascades (q = 0.011) with mutations from both UC and CD derived crypts contributing to the enrichment (Figures S5D).

## Discussion

We have used whole-genome sequencing of individual colonic crypts to provide the most accurate characterization of the somatic mutation landscape of the IBD-affected colon to date. The increased cancer risk of IBD patients results from a combination of higher mutation rate, particularly of deleterious mutations such as indels, from increased opportunity for clones carrying driver mutations to spread outside the confines of the crypt and from changes to the selective environment of the mucosa. Our results suggest that somatic substitution rate of the mucosa is accelerated 2.4-fold in disease, and the indel rate is accelerated as much as 7- fold. This increase is mostly driven by acceleration of single base substitution signatures 1, 5, and 18 and indel signatures 1 and 2, which are ubiquitous in the IBD-unaffected colon and are associated with cell proliferation and metabolic stress. The relatively large increase in the indel signatures suggests that IBD affected cells are particularly prone to DNA polymerase slippage during replication. Driver mutations are enriched in indels and the relatively high indel burden seen in IBD may help explain the increased risk of neoplasia, although this could not be directly tested in the present study.

Metabolic stress also results in an increased burden of somatic structural variants, which nevertheless remain rare in the IBD-affected mucosa. Structural variants are common in colorectal cancers and thus rapid increase in structural variation may be a hallmark of neoplastic transition, in line with previous reports (Baker et al., 2019). Increase in structural variation from healthy tissue to non-neoplastic disease has also been observed in liver disease (Brunner et al., 2019).

Colitis-associated colorectal cancers commonly arise from a background of large clonal fields (Choi et al., 2017). In our sample of non-dysplastic tissue, we find millimeter scale clonal expansions, although we note that for many inflamed regions, only a single small biopsy is available that limits our ability to detect large clones. *TP53* and *KRAS* mutations are thought to be key events in clonal spread in the IBD mucosa, but although we do observe a number of canonical cancer driver mutations in genes including *TP53* and *KRAS*, only *ARID1A* and *FBXW7* show significant evidence of positive selection.

Although there is substantial overlap in the driver landscape of IBD and non-IBD colon, important differences also exist. Our findings of enrichment of mutations in *PIGR*, *ZC3H12A*, and in the IL-17 and TLR pathways suggest there are distinct selection mechanisms in the colitis-affected colon, and somatic mutations potentially play a causal role in the pathogenesis of IBD. While this work was under review, two studies of somatic mutations in UC patients from the Japanese population were published that confirm our findings of positive selection of mutations in *ARID1A*, *FBXW7*, *PIGR*, *ZC3H12A*, and in the IL-17 pathway (Kakiuchi et al., 2020; Nanki et al., 2020). Importantly, our study shows that the same selective pressures are operative in mucosal tissue in both UC and CD.

The two papers also report mutations in additional genes including *NFKBIZ*, *IL17RA*, *TRAF3IP2*, and *NOS2*. We performed restricted-hypothesis testing of a set of 13 genes reported in these other two papers and replicated six at q < 0.05 (Table S5F). Importantly, the enrichments of truncating mutations we observe in the IL-17 and TLR pathways, which share many genes in common, are not driven by the genes discussed above because *PIGR*, *ZC3H12A*, *NFKBIZ*, and *NOS2* are not part of these pathways (according to Reactome), and no mutations were found in *TRAF3IP2.* This suggests that additional positively selected genes related to IL-17 and TLR signaling may be discovered in the IBD colon as sample size is increased. The difference in the number of *NFKBIZ* mutant crypts between the studies is noticeable. We detected only 3 truncating mutations in *NFKBIZ*, which is the most commonly mutated gene in Kakiuchi et al. (2020). This is reminiscent of our previous description of how selection of *NOTCH2* mutants in normal skin may vary between individuals of European and South Asian ancestry (Martincorena et al., 2015). Together with our observation that distinct mutations in the same gene are often found in crypts from the same individual, this leads us to speculate that differences in local environment or a person’s genetic background affects the strength of selective advantage posed by somatic variants and studies with larger sample sizes may be able to detect those interactions. We also observe smaller clones than those reported by Kakiuchi et al. (2020), who document clones spanning many centimeters in surgically resected colons. We speculate this may be due to patients undergoing colectomies having a more severe disease or due to different selection pressures between populations as mentioned above. Kakiuchi et al. (2020) also argue that some IBD-associated mutations, in particular in *NFKBIZ*, may prevent neoplastic transformation, but our data neither support nor refute this.

In their study, Nanki et al. (2020) show how IL-17A may be cytotoxic to epithelial cells and argue that clones carrying IL-17 pathway mutations are able to avert this cytotoxicity and thereby selectively expand in the inflamed environment. This has implications for the direction of effect of these mutations on IBD pathogenesis, because selective pressure would only be asserted following disease onset as Th17 cells infiltrate the tissue and secrete IL-17A in the vicinity of the epithelium. However, it could also be hypothesized that these mutations play a causal role in the pathogenesis of IBD through an effect on dysbiosis. Indeed, the discovery by Nanki et al. (2020) that *PIGR* mutations do not confer upon cells survival advantage in the presence of IL-17A may add weight to this hypothesis. Although *ZC3H12A* and *NFKBIZ* are involved in IL-17 signaling, both are also induced downstream of TLRs (Yamamoto et al., 2004; Matsushita et al., 2009) where they regulate the transcriptional changes that follow TLR signaling. Disruption of the IL-17 pathway itself may also play a causal role in the disease, because intestinal epithelial cell-specific knockout of components of the IL-17 pathway in mice results in commensal dysbiosis through downregulation of *Pigr* and other genes (Kumar et al., 2016). Thus, a positive feedback loop may be established, leading to ever greater spread of a pathogenic clone. It is worth noting that clinical trials of secukinumab and brodalumab (anti-IL-17A and anti-IL-17RA antibodies, respectively) for the treatment of CD have been carried out but either show no efficacy over placebo or worsen the disease (Hueber et al., 2012; Targan et al., 2016). Case reports of IBD in psoriasis patients receiving ixekizumab, a second IL-17A antibody, have been reported (Philipose et al., 2018; Smith et al., 2019), although post hoc analyses of ixekizumab trials suggest that IBD is a rare adverse outcome (Reich et al., 2017).

Our understanding of somatic evolution in normal tissues has improved greatly over the last few years but how and if somatic evolution contributes to the pathogenesis of complex traits other than cancer remains poorly understood. Clonal hematopoiesis has been associated with coronary heart disease (Jaiswal et al., 2014), and our work suggests that somatic evolution in the colonic mucosa may initiate, maintain, or perpetuate IBD. Large-scale analyses of cancers have started to reveal common themes of cancer evolution across tissues (Gerstung et al., 2020), and extending this work to other tissues exposed to chronic inflammation may similarly reveal patterns of remodeling of the selection landscapes associated with disease, but which need not drive neoplastic growth. Comparing the evolutionary forces in the IBD mucosa with those operating in psoriasis, celiac disease, asthma, and other diseases affecting epithelial cells is an area of special interest.

## STAR★Methods

### Key Resources Table

**Table:** 

REAGENT or RESOURCE	SOURCE	IDENTIFIER
**Deposited Data**		

BAM files containing sequencing data from crypts isolated from IBD patients.	This paper.	European Genome-phenome Archive (EGA) - accession code EGA: EGAD00001006061
Images of microdissections and physical distances between crypts.	This paper.	Mendeley Data: https://doi.org/10.17632/x3vsxpspn4.2
BAM files containing sequencing data from crypts isolated from controls.	Lee-Six et al., 2019	European Genome-phenome Archive (EGA) - accession codes EGA: EGAD00001004192 and EGA: EGAD00001004193
PCAWG mutational signatures.	Alexandrov et al., 2020	https://cancer.sanger.ac.uk/cosmic/signatures

**Software and Algorithms**		

Algorithms and software for calling somatic substitutions - CaVEMan.	Cancer Genome Project, Wellcome Trust Sanger Institute	http://cancerit.github.io/CaVEMan/
Algorithms and software for calling somatic indels - PIndel.	Cancer Genome Project, Wellcome Trust Sanger Institute	http://cancerit.github.io/cgpPindel/
Algorithms and software for calling somatic retrotranspositions - TraFiC-mem.	Rodriguez-Martin et al., 2020	https://gitlab.com/mobilegenomesgroup/TraFiC
Algorithms and software for calling somatic copy number changes - BRASS.	Cancer Genome Project, Wellcome Trust Sanger Institute	https://github.com/cancerit/BRASS
Algorithms and software for calling somatic copy number changes - ASCAT.	Cancer Genome Project, Wellcome Trust Sanger Institute	https://github.com/cancerit/ascatNgs
Algorithms and software for constructing phylogenetic trees - MPBoot.	Hoang et al., 2018	http://www.iqtree.org/mpboot/
Algorithms and software for extracting mutational signatures - HDP.	Roberts, 2018	https://github.com/nicolaroberts/hdp
Algorithms and software for estimating dN/dS ratios.	Martincorena et al., 2017	https://github.com/im3sanger/dndscv
Custom scripts documenting all other analyses.	This paper.	https://github.com/Solafsson/somaticIBD

### Resource Availability

#### Lead Contact

All requests for data and resources should be directed to the Lead Contact, Dr. Carl Anderson (ca3@sanger.ac.uk).

#### Materials Availability

This study did not generate new unique reagents or other materials.

#### Data and Code Availability

All sequencing data for the IBD cohort is available via the European Genome Phenome (https://ega-archive.org/). The accession number for the IBD data reported in this paper is EGA: EGAD00001006061. The accession numbers for the control data are EGA: EGAD00001004192 and EGA: EGAD00001004193. Phylogenetic trees, pileup read counts, histology images and physical distances between dissected crypts have been deposited to Mendeley data: https://doi.org/10.17632/x3vsxpspn4.2.

Custom scripts for carrying out the analyses described herein, including fitting of mixed-effect models, selection analyses and signature extraction, can be found under https://github.com/Solafsson/somaticIBD.

### Experimental Model and Subject Details

Colonic pinch-biopsies were donated by IBD patients undergoing regular surveillance of their disease at Addenbrooke’s hospital, Cambridge (Table S1). All samples were obtained with informed consent of the donor and the study was approved by the National Health Service (NHS) Research Ethics Committee (Cambridge South, REC ID 17/EE/0338) and by the Wellcome Trust Sanger Institute Human Materials and Data Management Committee (approval number 17/113). We have complied with all relevant ethical regulations.

All donors are of white-European ancestry. The time between clinical diagnosis and date of biopsy was used to define the disease duration of a given individual. We added six months to this number for all patients because symptoms often precede diagnosis by several months and to avoid setting the disease duration to zero for patients who donated samples at the time of diagnosis. Time of purine treatment was estimated by consulting electronic health records from NHS databases. Biopsies were annotated as never, previously or actively inflamed using all available clinical data and NHS histopathology archives. The biopsy images (or an image of a second biopsy from the same site of the colon) were reviewed by a histopathologist. None of the patients had colorectal cancer, adenoma or dysplasia. Patients who donated grid biopsies were chosen at random from those who had been diagnosed with IBD for more than a year, so to allow time for clonal spread to occur.

Biopsies from patients 1-26 were embedded in optimal cutting temperature (OCT) compound and sectioned, stained and fixed as previously described (Lee-Six et al., 2019). None of the samples were fixed in formalin. Subsequent biopsies were embedded in paraffin because this better preserved the morphology of the tissue. Biopsies were sectioned (10-20 μm), fixed to 4 μm PEN membrane slides (11600288, Leica) and stained with hematoxylin and eosin. Crypts were dissected using laser capture microdissection microscopy (LMD7000, Leica) and lyzed using ARCTURUS PicoPure DNA extraction kit (Applied Biosystems) according to the manufacturer’s instructions. DNA libraries were prepared as previously described (Lee-Six et al., 2019).

The control cohort was obtained from our previous publication on somatic mutations in the normal colon (Lee-Six et al., 2019). It consists of seven deceased organ donors, 31 individuals who underwent colonoscopy following a positive faecal occult blood test in a screening program (16 of which were not found to have an adenoma or a carcinoma and 15 of which had colorectal carcinoma, although the biopsies used were distant from these lesions) and three pediatric patients who underwent colonoscopy to exclude IBD and who were found to have a histologically and macroscopically normal mucosa. We excluded one subject from the control cohort who had undergone chemotherapy and was a clear outlier in terms of mutation burden and showed an abnormal mutation profile.

We note that the mutation burden of the pediatric subjects is what would be expected given the mutation burden observed in the other patients in the cohort, and that the crypts dissected from cancer patients did not show a higher mutation burden, distinct mutational processes or distinct driver landscape from biopsies donated from patients without cancer.

### Method Details

#### Genome sequencing

Samples from patient 1 through 19 (Table S1) were whole genome sequenced on Illumina XTEN® machines as previously described (Lee-Six et al., 2019). Samples from other patients were whole genome sequenced on Illumina Htp NovaSeq 6000® machines using 150bp, paired end reads except for patients 60-62, which were whole exome sequenced on the same platform using the Human All Exon V5 bait set. Reads were aligned to the human reference genome (NCBI build37) using BWA-MEM.

### Mutation calling and filtering

#### Substitutions

Base substitution calling was carried out in four steps: Discovery, filtering of the discovery set, genotyping and filtering of the genotypes. Mutations were first called using the Cancer Variants through Expectation Maximization (CaVEMan) algorithm (Jones et al., 2016). CaveMan uses a Bayesian classifier, incorporating base quality, read position, read orientation and more, to derive a posterior probability of all possible genotypes at every candidate site. Out of concern for field cancerization effect, patients 1 through 26, and patients from which only a few crypts were sequenced, were analyzed using a matched normal sample dissected from non-epithelial tissue from one of the biopsies. As it became apparent that clones did not stretch between biopsies, we stopped sequencing non-epithelial tissue control samples from patients if crypts were dissected from multiple biopsies.

The substitution calls were next filtered, as previously described (Lee-Six et al., 2019), to remove mapping artifacts, common single nucleotide polymorphisms and calls associated with the formation of cruciform DNA structures during library preparation. When matched normal samples were unavailable for the calling (see above), a large number of rarer germline variants remained post filtering. All sites where a somatic mutation was called in any crypt from a given patient were subsequently genotyped in all other samples from that patient by constructing read pileups and counting the number of mutant and wild-type reads. Only reads with a mapping quality of 30 or higher, and bases with a base quality of 30 or higher, were counted.

We next performed an exact binomial test to remove germline variants. True heterozygous germline variants should be present at a variant allele frequency (VAF) of 0.5 in all samples from an individual. Across all samples from a given individual, we aggregated variant and read counts at sites where a single nucleotide variant was called in at least one sample. We then used a one-sided exact binomial test to distinguish germline variants from somatic variants. The null hypothesis was that germline variants were drawn from a binomial distribution with a probability of success of 0.5, or 0.95 for the sex chromosomes in men. The alternative hypothesis was that these variants were drawn from distributions with a lower probability of success. The resulting p values were corrected for multiple testing using the Benjamini-Hochberg method. A variant was classified as somatic if q < 10^−3^, or q < 10^−2^ if fewer than five crypts had been dissected for the patient. For variants classified as somatic, we fitted a beta-binomial distribution to the number of variant supporting reads and total number of reads across crypts from the same patient. For every somatic variant, we determined the maximum likelihood overdispersion parameter (ρ) in a grid-based way (ranging the value of ρ from 10^−6^ to 10^-0.05^). A low overdispersion captures artifactual variants because they appear to be randomly distributed across samples and can be modeled as being drawn from a binomial distribution. In contrast, true somatic variants will be present at a VAF close to 0.5 in some, but not in all crypt genomes, and are thus best represented by a beta-binomial with a high overdispersion. To distinguish artifacts from true variants, we used ρ = 0.1 as a threshold, below which variants were considered artifacts. The code for this filtering approach is an adaptation of the Shearwater variant caller (Gerstung et al., 2014). Finally, we filtered out variants that were supported by fewer than three reads or where the sequencing depth was less than five.

#### Indels

Short deletions and insertions were called using the Pindel algorithm (Ye et al., 2009). We applied the same restrictions on median VAF and read counts as for substitutions, and germline indel calls were filtered using the same binomial filters as described above.

#### Sensitivity analysis

To estimate sensitivity we dissected and sequenced five crypts twice. Assuming the same sensitivity in both samples, a maximum likelihood estimate for the sensitivity when mutations not present in either sample go unobserved is:

$$S=\frac{2\times n_{2}}{n_{1}+2\times n_{2}}$$

Where n_2_ is the number of mutations called in both samples and n_1_ is the sum of mutations called in only one sample. As sensitivity depends on coverage, which is uneven for the members of a pair, this estimate should be considered to be a lower bound.

We compared the sensitivity estimates for our five biological duplicates with internal sensitivity estimation for CaveMan (Figure S2C). This used 170 samples from the same individual sequenced to varying depths and, to remove the effect of clonality of the sample, estimated the sensitivity for calling heterozygous germline variants in these samples.

Our samples are expected to have slightly lower sensitivity than this estimate for the following reasons:

1 The curve assumes perfect clonality (median VAF of 0.5), but the median-median VAF in the IBD and control cohorts is 0.44.
2 The curve doesn’t capture indels, for which sensitivity is expected to be slightly lower than for substitutions.
3 To increase specificity, this paper required a coverage of 5 and at least 3 reads supporting the mutation, while standard for CaveMan is coverage of 4 and 2 mutant reads.

#### Constructing phylogenetic trees

We used the MPBoot software (Hoang et al., 2018) to create a phylogenetic tree for each patient. MPBoot uses ultrafast bootstrap approximation to generate a maximum parsimony consensus tree. We assigned mutations to branches using a maximum likelihood approach, removing mutations which didn’t adhere to the tree structure (p < 0.01, maximum likelihood estimation).

#### Structural variants

Copy number variants were called using the BRASS algorithm (https://github.com/cancerit/BRASS) as previously described (Lee-Six et al., 2019). Calls were filtered using AnnotateBRASS (https://github.com/MathijsSanders/AnnotateBRASS) as previously described (Moore et al., 2020). When a matched normal sample was not available for a patient, we used a clonally unrelated sample from the same individual to filter germline variants. All variants passing filters were manually reviewed in a genome browser. For discovery of deletions at fragile sites of the genome, we manually reviewed the three regions in all the genomes.

Somatic retrotranspostions were called using TraFic algorithm (Rodriguez-Martin et al., 2020). Somatic events supported by read clusters without exact breakpoints were also included. To further identify somatic transduction events, translocation calls (i.e., read clusters) related with known L1 germline sources (Rodriguez-Martin et al., 2020) from BRASS algorithm were examined. All somatic retrotransposition events were manually reviewed.

Chromosome aneuploidies and deletions or duplications affecting large areas of chromosomes or whole chromosome arms were called using the ASCAT algorithm (Van Loo et al., 2010; Raine et al., 2016).

### Quantification and Statistical Analysis

#### Mutation rate comparisons between IBD patients and controls

Any test for a difference in mutation burden between cohorts must take into account all factors, biological and technical, which correlate with disease and/or affect mutation calling sensitivity. For our comparison of IBD and normal, we fitted linear mixed effects models taking the following factors into account:

1 Age is the most important predictor of mutation burden and the age distribution of the two cohorts is different. We included a fixed effect for age in the models to account for this.
2 Mutation burden differs for different sectors of the colon (Lee-Six et al., 2019). The IBD cohort is enriched with samples from the left side, as this is the area predominantly affected in UC patients. We included a fixed effect for location within the colon to account for this.
3 Observations are non-independent. We included in the models random effects for patient and for biopsy, with the random effect for biopsy nested within that for the patient.
4 Most embryonic mutations will be filtered as germline so at birth the mutation count is near zero. We therefore did not include a random intercept in the models but constrained the intercept to zero. The biological interpretation of this is that there are no somatic mutations present at birth.
5 The between-patient variance is likely greater in the IBD cohort as patients vary in the duration, extent and severity of their disease. The within-patient variance is also likely greater in the IBD cohort as biopsies taken from different sites of the colon vary in their disease exposure, number and duration of flares etc. To model this, we constructed a general positive-definite variance-covariance matrix for the random effects of patient and biopsy by cohort.
6 Any difference in the clonality of the colon between IBD patients and controls will affect the relative sensitivity to detect somatic mutations. To account for this, we adjusted the branch lengths of the phylogenetic trees and used the adjusted mutation counts as the response variable in the models. The adjustment was carried out as follows. Mutations with low variant allele frequencies (VAFs) will be missed at low coverage. Therefore, for each crypt, we first fitted a truncated binomial distribution to the VAF distribution of the crypt to estimate the true underlying median VAF (this is different from 0.5 because recent mutations may not yet have been fixed in the stem cell niche, and because of contamination of lymphocytes and other cells from the lamina propria, which do not carry the same somatic mutations as the epithelial cells). We next simulated 100,000 mutation call attempts by drawing the coverage of each call from a Poisson distribution, with the lambda set as the median coverage of the sample, and multiplying that with the median VAF estimate from the truncated binomial. The resulting value represents the number of reads that carry the mutated allele. We calculated sensitivity for the sample, S_s_, as the fraction of draws that resulted in four or more mutant reads, which is the number required by CaVEMan to call a mutation. The sensitivity of a branch with n daughter crypts, S_b_, was then calculated as:

$$S_{b}=1-\left(1-S_{s1}\right)*...*\left(1-S_{si}\right)*...*\left(1-S_{sn}\right).$$

The adjusted mutation count is thus the observed mutation count divided by the sensitivity of the branch. In this way, the mutation count of clones formed of stand-alone crypts is augmented more than that of branches with multiple daughter crypts. Even after these steps, a small but significant effect of coverage remained and a fixed effect for coverage was included in the models.

We compared models thus fitted with ones which additionally included disease duration as a fixed effect using a likelihood ratio test. The disease durations for never inflamed regions of the colons of IBD patients were set to zero.

As comparatively few structural variants are found in the dataset, we used Poisson regression within a generalized linear mixed effects framework to test for differences in structural variant number between cases and controls. We included the same random and fixed effects described above for base substitutions and indels and compared models with and without disease duration using a likelihood ratio test. The above statistical tests are two-sided as are all statistical tests performed in this manuscript. Full details and outputs of all statistical models used in this work are available in an R-markdown file accompanying the submission.

#### Mutational signature extraction and analyses

Define a mutational signature as a discrete probability distribution over a set of categorical mutation classes (for example, 96 classes for single base substitutions - according to the identity of the pyrimidine-mutated base pair, and the base 5′ and 3′ to it, see Alexandrov et al. (2013), 2020). We extracted mutational signatures using a hierarchical Dirichlet process (Roberts, 2018) (HDP, see the hdp R package https://github.com/nicolaroberts/hdp). This has the advantage of allowing simultaneous fitting to existing signatures and discovery of new signatures. We pooled the control and the IBD data and extracted signatures from both datasets as previously described for indels and single base substitutions separately (Lee-Six et al., 2019). We mapped mutations to branches of a phylogenetic tree and treated each branch with more than 50 mutations as a sample. We used signatures reported in colorectal cancer as priors and also included as priors signature 32, which is attributed to azathioprine therapy (Alexandrov et al., 2020), and signature 35, attributed to platinum-based chemotherapy, as there are patients in our cohort with history of using these drugs. Using the PCAWG terminology (Alexandrov et al., 2020), the priors used were SBS1, SBS2, SBS3, SBS5, SBS13, SBS16, SBS17a, SBS17b, SBS18, SBS25, SBS28, SBS30, SBS32, SBS35,SBS37, SBS40, SBS41, SBS43, SBS45 and SBS49 for substitutions and ID1, ID2, ID3, ID4, ID5, ID6, ID7, ID8, ID10 and ID14 for indels.

We used expectation maximization to deconvolute the HDP components into known PCAWG signatures. The cosine similarity between the HDP component corresponding to SBS1 was < 0.95 and we used expectation maximization to break the component down into PCAWG signatures. We then reconstituted the components using only those PCAWG signatures that accounted for > 10% of the mutations (this was done so as to avoid overfitting). This helped resolve the correlation between SBS1, SBS5 and SBS18. We merged components corresponding to SBS5 and SBS40 under the name of SBS5, as both are flat and difficult to distinguish. No other components had cosine similarity < 0.95 with their corresponding signatures and other PCAWG signatures accounting for > 10% of the mutations.

#### Selection analyses

To search for mutations under positive selection, we used the dNdScv method (Martincorena et al., 2017). We included never inflamed samples from the IBD cohort in the analysis as some uncertainty existed regarding the annotation of a handful of never-inflamed biopsies and we estimated that our analysis would suffer more from potential exclusion of drivers than from inclusion of more neutral mutations. We used the Benjamini-Hochberg method to correct for multiple testing.

To look for enrichment of mutations in pathways we defined 15 gene-sets (Table S6) as follows. We included all genes found to be under selection in colorectal cancer (Priestley et al., 2019) and a list of genes significant in a pan-cancer analysis of solid tumors (Priestley et al., 2019). We also chose a set of cellular pathways known to be important in IBD pathogenesis and epithelial homeostasis. The Reactome database was used to define the pathways (Fabregat et al., 2018), see Table S6 for accession dates and Reactome IDs of pathways. We chose the cytokine pathways TNF-Signaling, TNFR2, IL6, TGFb and IL17 for testing. We also defined a combined list of cytokines which included all of the above as well as IFNg, IL10, IL20, IL23, IL28, and IL36. We also decided to test other pathways shown by us and others through genome-wide association studies to be important in IBD pathogenesis (Jostins et al., 2012; de Lange et al., 2017). These were Toll-like receptor cascades, NOD-signaling, autophagy, unfolded protein response and epithelial cell-cell junctions. We included the PIP3/AKT signaling pathways as it is downstream of many of the pathways defined above and we had discovered two large scale deletions affecting genes in this pathway before performing the analysis. Finally, we defined a list of genes known to cause early-onset, monogenic forms of IBD. Many of the genes defined in the literature affect myeloid cell development and cause severe immunodeficiencies (Uhlig, 2013; Uhlig et al., 2014). We restricted our analysis to the union of monogenic-IBD genes which either are specifically thought to affect epithelial cells or were members of any of the pathways above.

We extracted global dN/dS values for missense and truncating variants for each of the 15 pathways/gene sets (Figure 6C; Table S6) separately and used the Benjamini-Hochberg method to correct for a total of 30 tests.

## Supplemental Information

Table S1. Cohort Characteristics, Related to Figures 1, 2, 3, 4, 5, and 6 Patient meta-data for IBD patients and the control cohort.

Table S2. Crypt Annotations, Meta-Data, and Mutation Burden, Related to Figures 1, 2, and 3

Table S3. Mutation Catalogs, Related to Figures 3 and 6 (A) All coding substitutions and indels in the IBD cohort. (B) All coding substitutions and indels in the control cohort. (C) All copy number variants called in both the IBD and control cohort. (D) Somatic retrotranspositions called in the IBD cohort. (E) Somatic retrotranspositions called in the control cohort.

Table S4. Driver Mutations, Related to Figure 6 (A) Canonical hotspot mutations in the Cancer Genome Atlas. (B) Putative drivers (superset of putative cancer drivers) found in cases and controls. List includes mutations found in genes with dN/dS > 1, loss-of-function mutations in known colorectal cancer genes and missense mutations occurring in canonical mutation hotspots associated with cancer development in the Cancer Genome Atlas. (C) Results of association analyses between putative cancer drivers and mutation burden and signature exposure.

Table S5. Results of the dNdS Analyses, Related to Figure 6 (A) Mutation counts and gene-level dNdS results for the IBD cohort. (B) Comparison of positive selection in healthy and the IBD-affected colon. (C) Results of pathway-level dNdS for missense and truncating mutations for the 15 pathways in Table S6 linked with IBD or colorectal cancer. Results are shown for UC, CD and controls. (D) Mutations in genes in the IL-17 signaling pathway. (E) Mutations in the toll-like receptor signaling pathway. (F) Restricted hypothesis testing of genes reported to be under positive selection in Kakiuchi et al. (2020).

Table S6. Gene Sets and Pathways Used in the dNdS Analysis, Related to Figure 6 Genes linked with colorectal cancer or pan-cancer or in pathways with a role in the pathogenesis of inflammatory bowel disease as defined in REACTOME.
